# Association Between Vertical Skeletal Pattern and Mandibular Cortical Morphology: A CBCT-Based Radiomorphometric Study

**DOI:** 10.3390/diagnostics16193174

**Published:** 2026-09-30

**Authors:** Buket Erdem, Mustafa Ozcan, Eda Didem Yalcin, Cagla Sar

**Affiliations:** 1 Department of Orthodontics, Faculty of Dentistry, Istanbul Health and Technology University, Istanbul 34180, Türkiye; mustafa.ozcan@istun.edu.tr (M.O.); cagla.sar@istun.edu.tr (C.S.); 2 Department of Dentomaxillofacial Radiology, Faculty of Dentistry, Istanbul Health and Technology University, Istanbul 34180, Türkiye; eda.yalcin@istun.edu.tr

**Keywords:** cone-beam computed tomography, mandibular cortical index, mental index, panoramic mandibular index, radiomorphometric indices, vertical skeletal pattern

## Abstract

**Background/Objectives**: Mandibular cortical morphology has been associated with vertical facial patterns, but differences in standardized radiomorphometric indices remain unclear. This study compared five mandibular indices among vertical skeletal patterns using CBCT-derived panoramic reconstructions. **Methods**: This retrospective cross-sectional study included 181 adults classified as hypodivergent (*n* = 42), normodivergent (*n* = 67), or hyperdivergent (*n* = 72) according to the sella–nasion-to-mandibular-plane angle. Mental (MI), panoramic mandibular (PMI), gonial (GI), antegonial (AI), and mandibular cortical (MCI) indices were assessed by a blinded dentomaxillofacial radiologist, and bilateral measurements were averaged. Continuous indices were compared using ANOVA or Welch ANOVA and ANCOVA adjusted for age and sex, and the MCI using the chi-square test and logistic regression. *p* values across the five indices were corrected using the Holm method. **Results**: No index differed significantly among the groups after correction for multiple comparisons. The MI showed the most notable nominal between-group difference (hypodivergent, 3.92 ± 0.73 mm; normodivergent, 3.73 ± 0.60 mm; hyperdivergent, 3.61 ± 0.68 mm). After adjustment for age and sex, the MI was 0.35 mm greater in hypodivergent than in hyperdivergent subjects (95% CI, 0.04–0.66), but the overall group effect did not remain significant after correction (*p* = 0.024; Holm *p* = 0.118). The unadjusted association between the MCI and vertical skeletal pattern (*p* = 0.035) did not remain significant after correction (Holm *p* = 0.174) or after adjustment. The PMI, GI, and AI showed no differences. **Conclusions**: After correction for multiple comparisons, vertical skeletal pattern was not significantly associated with any of the five radiomorphometric indices.

## 1. Introduction

Mandibular radiomorphometric indices provide qualitative and quantitative information on mandibular cortical morphology using panoramic radiographs or panoramic reconstructions [1]. Five commonly used indices are the mandibular cortical index (MCI), mental index (MI), panoramic mandibular index (PMI), gonial index (GI), and antegonial index (AI) [2,3,4,5]. The MCI, introduced by Klemetti et al. [2], qualitatively grades the porosity of the inferior mandibular cortex distal to the mental foramen. The MI, described by Ledgerton et al. [3], measures cortical thickness at the mental foramen, whereas the PMI, introduced by Benson et al. [4], expresses this thickness relative to the distance between the mental foramen and the inferior mandibular border. The GI and AI, described by Knezović Zlatarić et al. [5], measure cortical thickness at the gonial and antegonial regions, respectively. Collectively, these indices have been widely investigated as screening tools for reduced bone mineral density and osteoporosis risk. Although contemporary dentomaxillofacial radiology increasingly incorporates quantitative approaches for evaluating trabecular microarchitecture, conventional radiomorphometric indices remain widely used for assessing mandibular cortical morphology because of their standardized definitions, simplicity, and applicability to population-based studies. These indices primarily reflect the macroscopic cortical component of mandibular bone adaptation, whereas quantitative methods such as fractal dimension analysis provide complementary information on the internal trabecular structure [6].

Beyond their original application in osteoporosis screening, these radiomorphometric indices have been evaluated across various demographic and systemic conditions. Yalcin et al. [7] reported more porous MCI categories and lower MI and PMI values in patients with scleroderma than in healthy controls, whereas GI and AI did not differ significantly. Similar applications have been reported in patients with chronic kidney disease [8] and sickle cell disease [9]. Importantly, the indices do not appear to respond uniformly across populations or clinical conditions, suggesting that their discriminatory value may depend on the characteristic being evaluated.

Independently of systemic bone status, mandibular cortical morphology may also be influenced by local biomechanical loading [10]. Masticatory muscle characteristics and bite forces differ among vertical facial patterns [10,11,12,13], and corresponding differences in mandibular cortical bone have been reported. Tsunori et al. [14] found greater buccal and lingual cortical thickness in the molar region of short-faced than long-faced subjects, whereas Sato et al. [15] reported a negative association between mandibular molar cortical bone density and mandibular plane angle. Osato et al. [16] further demonstrated greater cortical width and PMI values in subjects with a low gonial angle, and Horner et al. [17] reported thicker cortical bone in hypodivergent than in hyperdivergent adults. Together, these findings support an association between vertical facial morphology and mandibular cortical characteristics.

Several CBCT-based studies have further examined cortical bone thickness across vertical facial types at specific anatomic sites. Sadek et al. [18] found significantly thinner cortical bone in high-angle subjects than in normal- and low-angle subjects at several interradicular sites, broadly consistent with earlier findings by Ozdemir et al. [19] and Swasty et al. [20]. However, differences across vertical facial types have not been uniformly observed across anatomic regions, suggesting regional variability in the relationship between vertical skeletal morphology and cortical bone thickness. Importantly, these studies have predominantly relied on site-specific linear measurements rather than standardized radiomorphometric indices. Although CBCT permits direct three-dimensional measurements, applying standardized two-dimensional radiomorphometric indices to CBCT-derived panoramic reconstructions provides a uniformly defined and reproducible approach for comparative evaluations while maintaining continuity with the extensive panoramic literature, including large-sample radiomorphometric studies [1,3] and large-cohort panoramic anatomical assessments [21]. In addition, CBCT-derived panoramic reconstructions reduce the effects of geometric magnification and anatomical superimposition associated with conventional panoramic radiography and allow standardized visualization of the mandibular cortex [22,23]. Whether radiomorphometric indices obtained from different mandibular regions similarly differ among vertical skeletal patterns therefore remains unclear.

To our knowledge, no previous study has evaluated the complete set of standardized radiomorphometric indices in individuals classified according to vertical skeletal pattern. Therefore, the aim of this study was to compare the mental index, panoramic mandibular index, gonial index, antegonial index, and mandibular cortical index on CBCT-derived panoramic reconstructions among individuals with hypodivergent, normodivergent, and hyperdivergent skeletal patterns. The null hypothesis was that none of the five indices would differ among the three vertical skeletal groups.

## 2. Material and Methods

### 2.1. Study Design and Sample

This retrospective cross-sectional study was conducted using CBCT records archived at the Department of Orthodontics and Department of Dentomaxillofacial Radiology, Istanbul Health and Technology University, Faculty of Dentistry, Istanbul, Turkey. The study protocol was approved by the Ethics Committee of Istanbul Health and Technology University (approval no. 2026/05-05) and was conducted in accordance with the Declaration of Helsinki.

CBCT scans previously obtained for diagnostic or treatment-planning purposes were retrospectively screened for eligibility. The inclusion criteria were age ≥20 and ≤35 years, absence of systemic diseases affecting bone metabolism, no history of orthognathic surgery or jaw trauma, and adequate image quality with clearly identifiable anatomic landmarks. CBCT scans showing pathologic lesions of the jaws or metallic artifacts obscuring the regions of interest were excluded. Subjects with a free-end edentulous area resulting from the absence of the most distal molar in any quadrant were also excluded; absence of third molars was not considered an exclusion criterion. Medical history was obtained from the hospital records, including the anamnesis forms completed at the time of imaging. The age range was restricted to 20–35 years to minimize the potential influence of residual craniofacial growth and age-related changes in mandibular cortical morphology [24,25]. The archive was searched for CBCT scans obtained between July 2023 and June 2026, yielding 428 potentially eligible records. Records were excluded sequentially, each according to the first criterion met: age outside the 20–35-year range (*n* = 128), a free-end edentulous area (*n* = 41), systemic diseases affecting bone metabolism (*n* = 38), pathologic lesions of the jaws (*n* = 21), history of orthognathic surgery (*n* = 5) or jaw trauma (*n* = 2), and inadequate image quality (*n* = 12). The final sample comprised 181 records.

### 2.2. Image Acquisition

CBCT images were acquired using a Planmeca ProMax 3D Mid unit (Planmeca Oy, Helsinki, Finland) with fields of view of 16 × 9, 16 × 16, or 20 × 10 cm, operating at 90 kVp and 12 mA, with an exposure time of 12–15 s. Images were reconstructed with an isotropic voxel size of 0.4 mm and analyzed using Planmeca Romexis Viewer software, Version 6.5.3 (Planmeca Oy, Helsinki, Finland) on a 24-inch UltraSharp LED TFT monitor (Dell Inc, Round Rock, TX, USA). Panoramic reconstructions were generated from each CBCT dataset using the panoramic reformatting module of Planmeca Romexis Viewer (Planmeca Oy, Helsinki, Finland), with individual arch tracing performed for each patient. Panoramic reconstructions were generated with an image thickness of 1 mm, and the reconstruction plane was oriented parallel to the Frankfort horizontal plane.

The same reconstruction protocol, including individual arch tracing, an image thickness of 1 mm, and a reconstruction plane parallel to the Frankfort horizontal plane, was applied to every record. Three fields of view were represented in the archive; the voxel size (0.4 mm) and reconstruction protocol were identical irrespective of the field of view, and in all included scans, the regions of interest were fully contained within the acquired volume, allowing bilateral measurement of all indices in every subject. In a previous CBCT study, repeated measurements of cortical bone thickness showed similarly high reliability at voxel sizes of 0.4 mm and 0.2 mm [26].

### 2.3. Skeletal Classification

Vertical skeletal pattern was determined using the angle between the sella–nasion plane and the mandibular plane (SN–MP angle) [27]. The SN–MP angle was measured on lateral cephalograms reconstructed from the same CBCT dataset using NemoCeph software, Version 20.10.0 (NemoTec, Madrid, Spain) by an orthodontist with 14 years of clinical experience (B.E.) who was independent of the radiomorphometric assessment. The use of CBCT-derived lateral cephalograms ensured that skeletal classification and radiomorphometric measurements were obtained from the same imaging session, eliminating any temporal discrepancy between records. Subjects were classified as hypodivergent (SN–MP < 27°), normodivergent (27° ≤ SN–MP ≤ 37°), or hyperdivergent (SN–MP > 37°), yielding 42, 67, and 72 subjects, respectively.

### 2.4. Radiomorphometric Measurements

**Figure 1:**
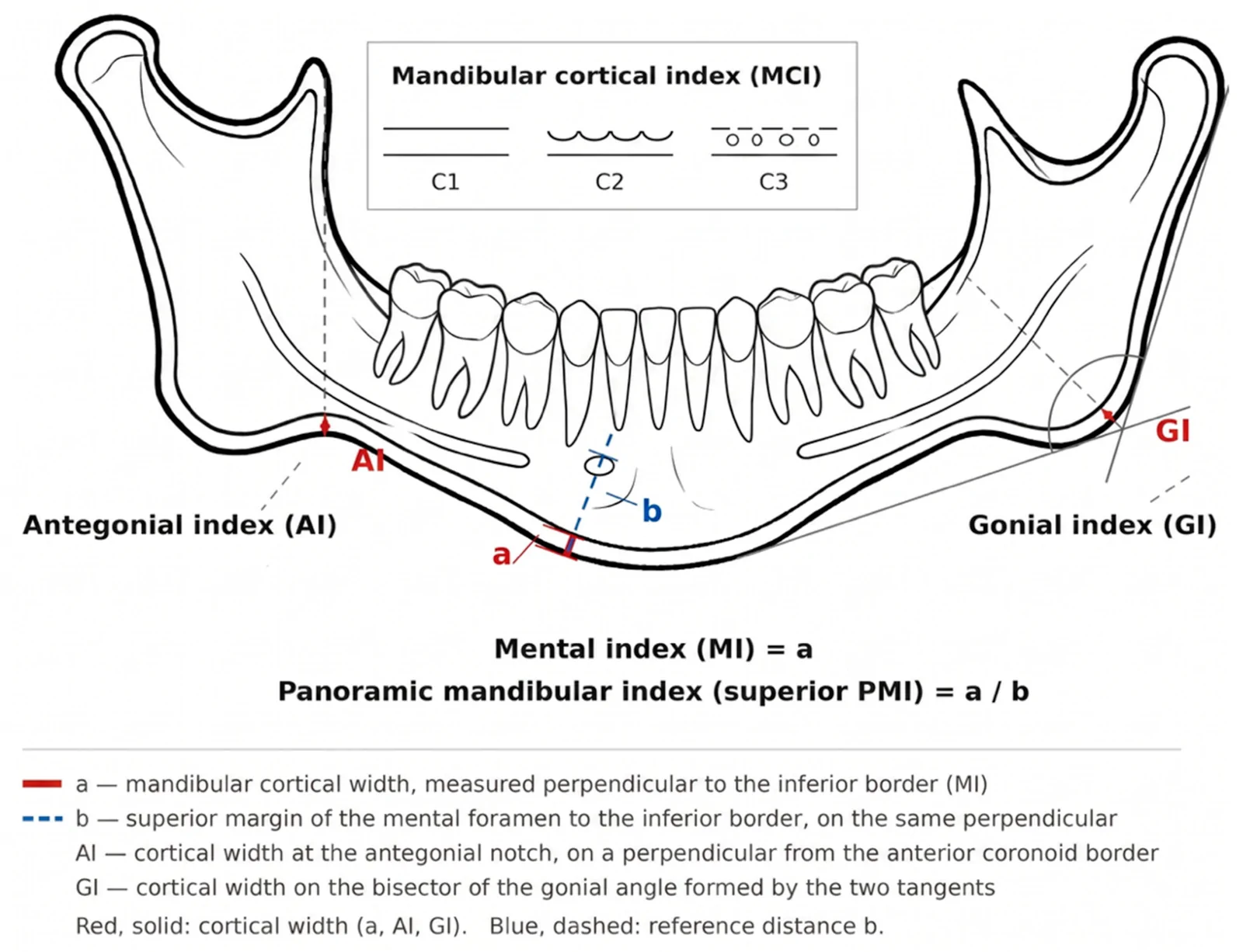
Schematic illustration of the mandibular radiomorphometric indices. The mental index (MI) represents mandibular cortical width (a) measured perpendicular to the inferior mandibular border at the mental foramen. The superior panoramic mandibular index (PMI) is calculated as the ratio of cortical width (a) to the distance from the superior margin of the mental foramen to the inferior mandibular border (b). The antegonial index (AI) represents cortical width at the antegonial region, and the gonial index (GI) represents cortical width measured along the bisector of the gonial angle. The mandibular cortical index (MCI) qualitatively classifies inferior mandibular cortical morphology as C1, C2, or C3 according to the degree of endosteal cortical erosion.

Five radiomorphometric indices were evaluated on CBCT-derived panoramic reconstructions, as defined in Table 1 and illustrated in Figure 1.

All measurements were performed directly on the panoramic reconstructions using Planmeca Romexis Viewer (Planmeca Oy, Helsinki, Finland). No magnification correction or external calibration was applied. The MI, GI, and AI were recorded as linear measurements, whereas the PMI was calculated as a ratio, with measurements obtained separately for the right and left sides. The MCI was assessed qualitatively by evaluating the morphology of the inferior mandibular cortex distal to the mental foramen bilaterally and was recorded as a single category (C1, C2, or C3) for each subject. When the MCI category differed between sides, the more severe cortical category was assigned.

The PMI was calculated using the superior margin of the mental foramen because the precision of the superior PMI has been reported to be approximately twice that of the inferior PMI [28]. All radiomorphometric assessments were performed by a dentomaxillofacial radiologist with 14 years of clinical experience (E.D.Y.) who was blinded to the subjects’ vertical skeletal classification.

### 2.5. Reliability of Measurements and Evaluations

Intrarater reliability was assessed in 20 randomly selected subjects (11% of the sample; hypodivergent, *n* = 3; normodivergent, *n* = 15; hyperdivergent, *n* = 2) after a 2-week interval. The SN–MP angle was remeasured by the same orthodontist (B.E.), and the MI, PMI, GI, and AI were remeasured by the same dentomaxillofacial radiologist (E.D.Y.) on one randomly selected side (right or left) per subject; the MCI was regraded bilaterally using the same criteria as in the first session. To avoid recall bias, both examiners were blinded to their initial measurements and MCI grades, to the order of the records, and to the group allocation of the subjects during the second session. Both sessions used the same CBCT datasets, software, reconstruction settings, and measurement protocols. Linear measurements were recorded to two decimal places.

Agreement between sessions was evaluated using the intraclass correlation coefficient (ICC; two-way mixed-effects model, absolute agreement, single measures) with 95% CIs for continuous variables and quadratic weighted kappa for the three-category MCI [29]. Systematic error was assessed with paired *t* tests for continuous variables and with the Stuart–Maxwell test of marginal homogeneity for the MCI, absolute measurement error was quantified using Dahlberg’s formula, and the minimal detectable change at the 95% confidence level (MDC95) for a single measurement was calculated as 1.96 × √2 × Dahlberg error. Because reliability was estimated from single-side measurements, these estimates are conservative for the bilateral mean values used in the main analyses.

ICC values were 0.96 (95% CI, 0.90–0.98) for the SN–MP angle, 0.91 (0.79–0.96) for the MI, 0.90 (0.77–0.96) for the PMI, 0.92 (0.81–0.97) for the GI, and 0.94 (0.85–0.98) for the AI; quadratic weighted kappa for the MCI was 0.96 (95% CI, 0.88–1.00). No systematic differences were found between sessions for the continuous measurements (all *p* > 0.05) or for the distribution of MCI grades (Stuart–Maxwell test, *p* > 0.05). Dahlberg errors were 0.91° for the SN–MP angle, 0.24 mm for the MI, 0.09 mm for the GI, 0.13 mm for the AI, and 0.016 for the PMI. The corresponding MDC95 values were 2.52° for the SN–MP angle, 0.67 mm for the MI, 0.25 mm for the GI, 0.36 mm for the AI, and 0.044 for the PMI.

### 2.6. Statistical Analysis

Categorical variables were summarized as frequencies and percentages and continuous variables as means and standard deviations. Differences in age and sex distribution among the three vertical skeletal groups were assessed using one-way ANOVA and the chi-square test, respectively.

Because the MI, PMI, GI, and AI were measured bilaterally, the relationship between side and vertical skeletal group was assessed before the primary analyses. Right and left measurements were moderately correlated (r = 0.51–0.66), and mixed-design ANOVA showed no significant main effect of side or group × side interaction for any index (all *p* > 0.05). Therefore, bilateral measurements were averaged to obtain a single value per subject for each continuous index.

Bilateral mean indices were compared among groups using one-way ANOVA, or Welch ANOVA when the assumption of homogeneity of variance (Levene test) was violated. Because only 6 subjects were classified as MCI category C3, categories C2 and C3 were combined (C1 vs. C2 + C3), and the association with vertical skeletal pattern was evaluated using the chi-square test with exact *p* values; the strength of association was quantified using Cramér V.

To account for potential confounding by age and sex, adjusted analyses were performed for all five radiomorphometric indices, irrespective of the results of the unadjusted comparisons. For continuous indices, ANCOVA was performed with age and sex as covariates; pairwise differences in adjusted means are reported with Bonferroni-adjusted 95% CIs and *p* values, and effect sizes as partial η^2^. For the dichotomized MCI, binary logistic regression was performed with the normodivergent group as the reference category; the overall effect of vertical skeletal pattern was evaluated using the likelihood-ratio test, and associations were expressed as ORs with 95% CIs.

The five indices were treated as a single family of hypotheses. Within both the unadjusted and adjusted analyses, the overall group *p* values were corrected for multiplicity using the Holm–Bonferroni method. For the four continuous indices, the corresponding ANOVA or ANCOVA F-test *p* values were used, whereas the chi-square test *p* value and the likelihood-ratio test *p* value were used for the unadjusted and adjusted analyses of MCI, respectively.

## 3. Results

A total of 181 CBCT scans were included, comprising 98 men (54.1%) and 83 women (45.9%), with a mean age of 28.94 ± 4.20 years (range, 20–35 years). Age did not differ significantly among the vertical skeletal groups (*p* = 0.172), whereas sex distribution differed significantly (*p* = 0.023), with a higher proportion of men in the hyperdivergent group (66.7%) than in the normodivergent (46.3%) and hypodivergent (45.2%) groups (Table 2).

Right and left measurements of the continuous indices were moderately correlated (r = 0.51–0.66). No significant main effect of side (*p* = 0.48–0.96) or group × side interaction (*p* = 0.15–0.99) was observed for any index; therefore, bilateral mean values were used in all subsequent analyses.

In the unadjusted analyses, none of the four continuous indices differed significantly among the vertical skeletal groups (Table 3). Mean MI was lower in the hyperdivergent group (3.61 ± 0.68 mm) than in the hypodivergent group (3.92 ± 0.73 mm), but the overall group difference did not reach statistical significance (*p* = 0.059; Holm *p* = 0.238).

One-way ANOVA, except †Welch ANOVA (Levene test *p* < 0.05). Holm *p*, corrected across the five indices (including the MCI, Table 4).

The distribution of the dichotomized MCI differed among the groups (χ^2^ = 6.775; exact *p* = 0.035; Cramér V = 0.193), with the highest proportion of C2 + C3 classification in the hyperdivergent group (66.7%); however, this association did not remain significant after correction for multiplicity (Holm *p* = 0.174) (Table 4).

Chi-square test with exact *p* value. Holm *p*, corrected across the five indices (including the continuous indices, Table 3).

After adjustment for age and sex, MI showed a nominal overall association with vertical skeletal pattern (F = 3.827; *p* = 0.024; partial η^2^ = 0.042), which did not remain significant after correction for multiplicity (Holm *p* = 0.118) (Table 5). Adjusted mean MI values were 3.93, 3.77, and 3.58 mm in the hypodivergent, normodivergent, and hyperdivergent groups, respectively. In pairwise comparisons, adjusted MI was 0.35 mm greater in the hypodivergent than in the hyperdivergent group (95% CI, 0.04–0.66; Bonferroni-adjusted *p* = 0.022), whereas the other pairwise differences were not significant. Because the multiplicity-corrected omnibus test was not significant, these pairwise results should be regarded as exploratory. PMI, GI, and AI showed no association with vertical skeletal pattern after adjustment (all Holm *p* = 1.000).

For MCI, vertical skeletal pattern was not associated with C2 + C3 classification after adjustment for age and sex (likelihood-ratio *p* = 0.194; Holm *p* = 0.775). Compared with the normodivergent group, neither the hyperdivergent (OR, 1.93; 95% CI, 0.92–4.04; *p* = 0.080) nor the hypodivergent group (OR, 1.65; 95% CI, 0.71–3.80; *p* = 0.241) showed significantly greater odds of C2 + C3 classification. Female sex was independently associated with lower odds of C2 + C3 classification (OR, 0.25; 95% CI, 0.13–0.47; *p* < 0.001).

Adjusted means estimated at the mean age and sex distribution of the sample. Pairwise differences are reported with Bonferroni-adjusted 95% CIs and *p* values. In the MI model, sex F = 4.860, *p* = 0.029; age F = 3.240, *p* = 0.074. Holm *p*, corrected across the five indices.

Overall contribution of vertical skeletal group tested by likelihood-ratio test (χ^2^ = 3.284; df = 2).

## 4. Discussion

An important methodological consideration of the present study is the use of CBCT-derived panoramic reconstructions rather than conventional panoramic radiographs. Conventional panoramic imaging is inherently affected by magnification, geometric distortion, and superimposition of anatomical structures, which may influence linear measurements of mandibular cortical morphology. CBCT-derived reconstructions reduce projection-related geometric distortion and anatomical superimposition, providing a more standardized basis for cortical measurements [22,23].

The principal finding of this study was that, after correction for multiple comparisons, none of the five radiomorphometric indices differed significantly among the vertical skeletal groups. The MI showed the most notable nominal between-group difference, with lower values in hyperdivergent than in hypodivergent subjects; this difference was nominally significant after adjustment for age and sex (adjusted difference, 0.35 mm) but did not remain significant after Holm correction. The PMI, GI, and AI showed no differences, and the unadjusted association between the MCI and vertical skeletal pattern did not persist after covariate adjustment or multiplicity correction. The null hypothesis was therefore not rejected, and the observed pattern should be regarded as exploratory.

Although it did not remain significant after correction, the direction of the MI difference is consistent with previous CBCT studies reporting thinner cortical bone in individuals with increased vertical facial dimensions. Horner et al. [17] reported generally thicker buccal cortices in hypodivergent than in hyperdivergent adults, and Ozdemir et al. [19] found significantly thinner cortical bone in high-angle subjects at several maxillary and mandibular alveolar sites. Sadek et al. [18] demonstrated that differences associated with vertical facial type were confined to selected interradicular regions, and Swasty et al. [20] found site-dependent differences in mandibular cortical thickness. The magnitude of the adjusted MI difference in the present study (0.35 mm) is comparable to the hypodivergent–hyperdivergent differences reported by Horner et al. [17] (0.08–0.64 mm) and Sadek et al. [18] (0.16–0.62 mm), although these studies evaluated different anatomical sites and measurement methods.

Differences in cortical thickness among facial types have been attributed to differences in functional loading. Hyperdivergent individuals generally exhibit lower masticatory muscle activity and bite forces, and cortical bone adapts to its mechanical environment [10,11,12,13]. A useful contrast is provided by clinical situations in which masticatory muscle activity is deliberately and abruptly reduced. Yuksel et al. reported a significant decrease in trabecular fractal dimension at the mandibular angle six months after botulinum toxin type A injection into the masseter muscle [6]. Although the time scale and mechanism differ from the constitutionally lower loading of hyperdivergent individuals, and the reported changes concerned trabecular rather than cortical bone, these findings support the biological plausibility of reduced masticatory loading being reflected in mandibular bone structure. Because masticatory muscle activity and bite force were not measured in the present study, this explanation remains a hypothesis, and the present findings do not provide direct evidence for it.

Right and left measurements showed neither a main effect of side nor a group × side interaction for any index. This is consistent with previous studies that reported no systematic side differences in cortical bone thickness or evaluated only one side [15,17,19,30]. Analyzing bilateral means therefore avoided treating correlated measurements from the same individual as independent observations, and the data did not support a side-specific effect.

Although the PMI incorporates the same cortical measurement used for the MI, it additionally normalizes cortical thickness to the distance between the mental foramen and the inferior mandibular border. In the present study, the PMI showed no difference among groups, whereas the MI showed the largest, although nonsignificant after correction, difference. Normalization to mandibular dimensions may attenuate differences in absolute cortical thickness, which could explain this discrepancy. Osato et al. [16] reported greater PMI values in subjects with a low gonial angle; however, their study used extreme gonial-angle groups and differed substantially in sample selection and analytical approach.

Neither the GI nor the AI differed among vertical skeletal groups. Together with the MI findings, these results do not demonstrate a true difference between anatomical regions: a nominal difference at one site and nonsignificant differences at other sites do not in themselves show that the sites differ, and the present study did not formally test a site × group interaction. Evidence from other mandibular regions suggests that the relationship between facial type and cortical thickness may vary by site; Lo Giudice et al. [31] found the opposite pattern at the mandibular condyle, with cortical thickness greatest in hyperdivergent and lowest in hypodivergent subjects, and other studies have reported site-dependent associations [32,33]. Whether radiomorphometric indices from different mandibular regions differ in their relationship with vertical skeletal pattern requires a study designed to test this question directly.

The MCI requires separate consideration because it represents cortical morphology rather than a direct linear measurement of cortical thickness. The unadjusted distribution of MCI categories differed among vertical skeletal groups, but this association did not remain significant after correction for multiplicity and was not present after adjustment for age and sex. Because the hyperdivergent group included a higher proportion of men and male sex was strongly associated with C2 + C3 classification, the unadjusted association appears to have been influenced by differences in sex distribution among the groups [34].

The magnitude of the observed difference should be interpreted in relation to the resolution and precision of the measurements. The adjusted difference in MI between the hypodivergent and hyperdivergent groups (0.35 mm) should be interpreted in the context of the measurement precision of the method. Although voxel size may influence measurement precision and partial-volume effects may affect cortical boundary delineation, voxel size should not be equated directly with measurement error [20]. However, this difference refers to group means, which were estimated with a standard error of 0.13 mm, and random measurement error of the magnitude observed in the reliability analysis (Dahlberg error, 0.24 mm) would be expected to attenuate rather than create differences between groups, particularly because the radiologist was blinded to group allocation. At the individual level, by contrast, the minimal detectable change for a single MI measurement was approximately 0.67 mm; a difference of 0.35 mm could therefore not be resolved reliably in an individual patient and should not be used for individual diagnosis or treatment planning.

From a clinical perspective, the present findings should be interpreted cautiously. The adjusted MI difference of 0.35 mm is a group-level estimate that did not remain significant after correction for multiple comparisons. In the context of osteoporosis screening, an MI of 3 mm or less has commonly been used as a referral threshold [1,22], whereas the adjusted mean MI values of all three groups (3.58–3.93 mm) were above this value; the observed difference would therefore not be expected to cross this commonly used screening threshold in this sample. A similar nonuniform pattern among mandibular radiomorphometric indices was reported by Ocak et al. [35], who found differences in the MI and PMI among sagittal malocclusion groups, whereas the GI, AI, and MCI did not differ. Cortical bone thickness has been associated with the primary stability and success of orthodontic mini-implants [36,37,38], with higher success rates reported when cortical thickness at the insertion site is at least 1 mm [37], and a recent CBCT study reported greater cortical bone width at symphyseal mini-implant insertion sites in low-angle than in average- and high-angle individuals [39]. However, the present study measured cortical thickness at the inferior mandibular border rather than at the alveolar sites where mini-implants are usually placed, and implant stability and clinical outcomes were not investigated. Any inference from these findings to skeletal anchorage would therefore be speculative, and local assessment of bone at the intended insertion site remains the appropriate basis for anchorage planning.

A methodological strength of this study was the standardized assessment of five radiomorphometric indices on CBCT-derived panoramic reconstructions by a blinded, experienced dentomaxillofacial radiologist, with high measurement reproducibility (ICC, 0.90–0.94 for the radiomorphometric indices; weighted κ, 0.96 for the MCI). Although these indices were originally developed for conventional panoramic radiographs, their assessment on CBCT-derived images is supported by previous imaging research. Koseoglu Secgin et al. [22] reported good agreement between CBCT and conventional panoramic imaging for MI measurements and improved visualization of the inferior mandibular cortex on CBCT-based images, particularly for the assessment of cortical porosity. Nevertheless, radiomorphometric values obtained from CBCT-derived panoramic reconstructions should not be assumed to be directly interchangeable with those obtained from conventional panoramic radiographs [23].

The radiomorphometric indices evaluated in this study are linear or ordinal descriptors that primarily reflect the macroscopic characteristics of the mandibular cortex. Quantitative image analysis approaches such as fractal dimension (FD), first-order statistics (FOS), and gray-level co-occurrence matrix (GLCM) texture analysis instead describe trabecular structural complexity, grayscale distribution, and spatial texture. FD analysis of dental radiographs has been evaluated in a systematic review and meta-analysis [40], and has been assessed alongside conventional radiomorphometric indices in the mandible [41], while Yuksel et al. [42] recently combined FD, FOS, and GLCM features to characterize bone architecture in grafted maxillary sinus regions on panoramic radiographs. Cortical radiomorphometric indices and texture-based parameters may therefore be regarded as complementary approaches, although texture analysis of CBCT images requires caution because CBCT gray values are not standardized Hounsfield units and may be influenced by acquisition and reconstruction parameters.

Several limitations should be acknowledged. The analyses were exploratory: no primary outcome was prespecified, five related outcomes were compared, and no outcome remained significant after correction for multiple comparisons; the findings therefore require confirmation in an independent sample with a prespecified primary outcome. The retrospective and cross-sectional design precludes causal inference, and the groups were not matched for sex, although age and sex were accounted for in the adjusted analyses. All radiomorphometric measurements were performed by a single observer, so inter-observer reproducibility was not assessed. Masticatory muscle activity, bite force, and chewing-side preference were not assessed; therefore, the proposed functional explanation remains inferential. In addition, the trabecular compartment was not evaluated. Although three fields of view were used during CBCT acquisition, the potential influence of field-of-view size on the radiomorphometric measurements was not independently evaluated and therefore cannot be completely excluded. Body mass index and smoking status were not available in the records, and residual confounding by these factors cannot be excluded. In addition, sagittal skeletal relationship was not assessed in the present study. Because sagittal malocclusion has been associated with differences in mandibular cortical indices [35], residual confounding by sagittal skeletal relationship cannot be excluded. Future prospective studies incorporating functional variables, site-specific cortical measurements, and quantitative trabecular assessment may further clarify the relationship between vertical skeletal morphology and mandibular bone architecture.

## 5. Conclusions

In this exploratory analysis of 181 adults, none of the five radiomorphometric indices differed significantly among vertical skeletal groups after correction for multiple comparisons. Among the evaluated indices, the MI showed the most notable nominal between-group difference, with a 0.35-mm lower value in hyperdivergent than in hypodivergent subjects after adjustment for age and sex; however, this finding did not remain significant after multiplicity correction and should therefore be considered exploratory.

The present data do not demonstrate a generalized difference in mandibular cortical morphology according to vertical skeletal pattern. Larger studies with prespecified analytical plans and quantitative assessment of both cortical and trabecular bone are warranted.

## Figures and Tables

**Table 1:** Definitions of the radiomorphometric indices evaluated in this study.

Index	Definition
Mental index (MI)	Thickness (mm) of the inferior mandibular cortex at the point of intersection of a line dropped perpendicularly from the midpoint of the mental foramen to the line tangent to the inferior mandibular border
Panoramic mandibular index (PMI)	Ratio of the cortical bone thickness below the mental foramen to the distance from the upper border of the mental foramen to the inferior mandibular border
Gonial index (GI)	Thickness (mm) of the mandibular cortex measured along the bisector of the gonial angle, formed by the tangent to the posterior border of the ramus and the tangent to the inferior border of the mandible
Antegonial index (AI)	Thickness (mm) of the cortical bone at the site where a line extending tangentially from the anterior border of the ascending ramus meets the inferior border of the mandible, measured perpendicular to the inferior border
Mandibular cortical index (MCI)	Qualitative classification of the inferior mandibular cortex distal to the mental foramen: C1, the endosteal margin is even and sharp on both sides; C2, the endosteal margin shows semilunar defects and/or endosteal cortical residues one to three layers thick on one or both sides; C3, the cortical layer forms heavy endosteal cortical residues and is clearly porous

**Table 2:** Demographic characteristics of the study sample overall and by vertical skeletal group. Sex distribution was compared by chi-square test; age was compared by one-way ANOVA. SD, standard deviation. * *p* < 0.05.

	Total (*n* = 181)	Hypodivergent (*n* = 42)	Normodivergent (*n* = 67)	Hyperdivergent (*n* = 72)
Sex, *n* (%)				
Male	98 (54.1)	19 (45.2)	31 (46.3)	48 (66.7)
Female	83 (45.9)	23 (54.8)	36 (53.7)	24 (33.3)
*p*	—		0.023 *	
Age, y				
Mean ± SD	28.94 ± 4.20	29.60 ± 3.80	28.19 ± 4.18	29.25 ± 4.39
Median (min–max)	29 (20–35)	30 (21–35)	28 (20–35)	29.5 (20–35)
*p*	—		0.172	

**Table 3:** Unadjusted comparison of the bilateral mean radiomorphometric indices among the vertical skeletal groups.

Index	Hypodivergent (*n* = 42)	Normodivergent (*n* = 67)	Hyperdivergent (*n* = 72)	F	*p*	Holm *p*
MI, mm	3.92 ± 0.73	3.73 ± 0.60	3.61 ± 0.68	2.868	0.059	0.238
PMI	0.332 ± 0.083	0.331 ± 0.059	0.315 ± 0.064	1.397	0.252 †	0.757
GI, mm	1.42 ± 0.44	1.44 ± 0.39	1.44 ± 0.31	0.025	0.976 †	0.976
AI, mm	2.79 ± 0.69	2.87 ± 0.65	2.70 ± 0.62	1.163	0.315	0.757

Values are means ± SD of the right and left measurements. MI, mental index; PMI, panoramic mandibular index; GI, gonial index; AI, antegonial index; SD, standard deviation. † refers to the use of Welch ANOVA due to significant Levene’s test results (*p* < 0.05).

**Table 4:** Distribution of mandibular cortical index categories among the vertical skeletal groups.

MCI	Hypodivergent (*n* = 42)	Normodivergent (*n* = 67)	Hyperdivergent (*n* = 72)	χ^2^	Exact *p*	Holm *p*
	*n* (%)	*n* (%)	*n* (%)			
C1	18 (42.9)	37 (55.2)	24 (33.3)	6.775	0.035	0.174
C2 + C3	24 (57.1)	30 (44.8)	48 (66.7)			

MCI, mandibular cortical index; Cramér V = 0.193.

**Table 5:** Age- and sex-adjusted analyses of the radiomorphometric indices: ANCOVA for the bilateral mean continuous indices (**A**), pairwise contrasts for the MI (**B**), and logistic regression for the dichotomized MCI (**C**).

A. ANCOVA (Adjusted Means)	Hypodivergent	Normodivergent	Hyperdivergent	F	*p*	Holm *p*	Partial η^2^
MI, mm	3.93	3.77	3.58	3.827	0.024	0.118	0.042
PMI	0.330	0.330	0.317	0.832	0.437	1.000	0.009
GI, mm	1.44	1.45	1.42	0.064	0.938	1.000	0.001
AI, mm	2.76	2.87	2.72	1.021	0.362	1.000	0.011
**B. Pairwise contrasts for the MI**		**Difference in adjusted means, mm (95% CI)**				**Bonferroni *p***	
Hypodivergent vs. hyperdivergent		0.35 (0.04 to 0.66)				0.022	
Normodivergent vs. hyperdivergent		0.19 (−0.09 to 0.46)				0.297	
Hypodivergent vs. normodivergent		0.16 (−0.15 to 0.48)				0.642	
**C. Logistic regression: MCI C2 + C3 vs. C1**		**OR (95% CI)**				***p***	
Vertical skeletal group (overall)		—				0.194 (Holm *p* 0.775)	
Normodivergent (reference)		1.00				—	
Hypodivergent		1.65 (0.71–3.80)				0.241	
Hyperdivergent		1.93 (0.92–4.04)				0.080	
Female sex (vs. male)		0.25 (0.13–0.47)				<0.001	
Age (per year)		1.06 (0.98–1.15)				0.127	

ANCOVA, analysis of covariance; CI, confidence interval; MCI, mandibular cortical index; OR, odds ratio.

## Data Availability

The data presented in this study are available on request from the corresponding author due to privacy restrictions.
